# Tandem duplication of a genomic region encoding glutathione S-transferase epsilon-2 and -4 genes in DDT-resistant *Anopheles stephensi* strain from India

**DOI:** 10.1038/s41598-022-21522-8

**Published:** 2022-10-25

**Authors:** Cherry L. Dykes, Gunjan Sharma, Abhisek K. Behera, Neera Kapoor, Mark J. I. Paine, Martin J. Donnelly, Om P. Singh

**Affiliations:** 1 National Institute of Malaria Research, Sector 8, Dwarka, New Delhi, 110077 India; 2 Indira Gandhi National Open University, Maidangarhi, New Delhi, 110068 India; 3 Department of Vector Biology, Liverpool School of Tropical Medicine, Liverpool, L3 5QA UK

**Keywords:** Evolution, Genetics, Molecular biology, Zoology

## Abstract

The glutathione S-transferases (GST) genes are a multigene family of enzymes involved in the metabolism of endogenous and xenobiotic compounds by catalysing the conjugation of the reduced form of glutathione to the substrate. The epsilon class of GST (GSTe), unique to arthropods, is known to be involved in the detoxification process of several classes of insecticides, and GSTe2 in particular is known to have DDT dehydrochlorinase activity. This communication reports a tandem duplication of a genomic region encoding GSTe2 and GSTe4 genes in a laboratory-colonized DDT-resistant *Anopheles stephensi.* We identified duplication breakpoints and the organization of gene duplication through Sanger sequencing performed on long-PCR products. Manual annotation of sequences revealed a tandemly-arrayed duplication of a 3.62 kb segment of GST epsilon gene clusters comprised of five genes: a partial GSTe1, GSTe2, GSTe2-pseudogene, GSTe4 and partial GSTe5, interconnected by a conserved 2.42 kb DNA insert segment major part of which is homologous to a genomic region located on a different chromosome. The tandemly duplicated array contained a total of two GSTe2 and three GSTe4 functional paralog genes. Read-depth coverage and split-read analysis of Illumina-based whole-genome sequence reads confirmed the presence of duplication in the corresponding region of the genome. The increased gene dose in mosquitoes as a result of the GSTe gene-duplication may be an adaptive process to increase levels of detoxifying enzymes to counter insecticide pressure.

## Introduction

*Anopheles stephensi* is an important malaria vector distributed in countries of South Asia, the Middle East and the Horn of Africa. In India, this species is regarded as an urban malaria vector that prefers to breed in clean water habitats, preferably in cemented tanks (overhand tanks, cisterns, fountains) and building construction sites^1^. In recent years, this species has gained global attention due to its expansion into territories where it was never reported before, such as the Lakshadweep islands of India^2^, Sri Lanka^3^, and countries of the Horns of Africa, viz. Djibouti^4,5^, Ethiopia^6,7^ and Sudan^8^. The spread of *An. stephensi* has been considered as a major potential threat to malaria control and elimination in urban areas by the World Health Organization (WHO). Consequently, WHO issued an alert to member-states of affected and surrounding countries to take immediate action including considerating the introduction of core control tools directed against adult mosquitoes, namely LLINs or IRS in areas where *An. stephensi* is found and these interventions are not already being used^8^.

*Anopheles stephensi* in India is generally resistant to commonly used insecticides^9^ and is developing resistance against pyrethroids^10^. The mechanisms of insecticide resistance in this species are, however, poorly studied. Two alternative knockdown resistance (*kdr*) mutations, L1014F and L1014S, have been reported in this species^11^ with the varied distribution of alleles in different parts of India^12^, Afghanistan^13^ and countries of the Horn of Africa^14,15^. Little is known about alternative physiological resistance that is achieved either by the overexpression of xenobiotic-metabolizing enzyme or through the altered affinity of the enzyme for the insecticide due to structural changes which facilitate a faster rate of detoxification of the insecticide^16^. Major enzyme families responsible for insecticide metabolization belong to cytochrome P450 monooxygenases cytochrome P450s (CYP450), carboxyl-cholinesterases and glutathione S-transferases (GSTs)^17^.

Glutathione S-transferases (GST) are a superfamily of isoenzymes involved in the cellular detoxification of both xenobiotic and endogenous compounds by catalysing the conjugation of the reduced form of glutathione (GSH) to the substrate. GSTs are reported to be involved in resistance against several groups of insecticides such as organophosphates, organochlorines and pyrethroid groups of insecticides^18^, either through direct involvement in metabolism/sequestration of insecticides or indirectly by protecting against oxidative stress induced by insecticide exposure^19,20^. Among several gene families of GST reported, epsilon (GSTe) and delta class of enzymes are specific to arthropods^21^, and are the largest classes of insect-GSTs implicated in resistance against all major classes of insecticides^18,22–24^. A cluster of six to eight GSTe ortholog genes has been identified in mosquitoes^25^, however, annotation of the whole genome sequence (WGS) of *An. stephensi* available at VectorBase (AsteS1.8, SDA-500 strain) revealed the presence of all known eight ortholog GSTe genes, namely, GSTe3, GSTe7, GSTe1, GSTe2, GSTe4, GSTe5, GSTe6 and GSTe8 arranged sequentially on the scaffold KB664467 (SDA-500) which maps on chromosome 3R (GenBank accession: CP032234.1). GSTe genes have been frequently implicated to be associated with resistance against organochlorines, organophosphates and pyrethroids^23,27–33^. GSTe2, in particular, has been demonstrated to have specific DDT-dehydrochlorinase activity^21,34^.

Several mechanisms of insecticide resistance have been reported, mainly altered target sites due to mutation/s which render them to be less sensitive to the insecticide, upregulation or overexpression of insecticide-metabolizing genes. A single amino acid change in GSTe2 is known to confer high levels of metabolic resistance to DDT in *Anopheles funestus*. Less is known about the role of gene duplication in the increased copy number of insecticide-metabolizing genes, which is a major driving force in the evolution of genomes and in the creation of genes with new functions and plays an important role in adaptation to changing environments^35^. In this communication, we report the tandemly-arrayed duplication arrangement of a genomic region encoding GSTe2 and GSTe4 in a DDT-resistant *An. stephensi* line leading to enhanced mRNA expression and gene diversification leading to multiple functional paralogous genes, which is most likely an adaptive mechanism to cope with stress caused by the insecticides through positive selection.

## Results

### Molecular characterization of *An. stephensi *GST-epsilon genes (*AsGSTe*) and evidences of gene duplication

Molecular characterization of GST-epsilon gene array, mainly of GSTe2 and GSTe4 which have been implicated associated with insecticide resistance^17,21,31,34^, was performed for the laboratory selected DDT-resistant (LT_50_: 14 h) strain originating from Alwar (Alw-R), DDT-highly susceptible individuals originating from Chennai (Che-S) and a preserved specimen from an old colony of *An. stephensi* from Delhi (Del) (colonized in the 1990s, possibly susceptible). The individuals from Chen-S strain (F_5_ generation) which were knocked down within 10 min of exposure with 4%DDT-impregnated paper, were used as ‘susceptible’ mosquitoes A long-PCR (L-PCR) strategy was employed to amplify a genomic region spanning *AsGSTe2,* pseudogene *ψAsGSTe2* and *AsGSTe4* through long PCR (L-PCR) which was successful in all strains, but when an extended genomic region spanning *AsGSTe1* through *AsGSTe5* (comprising *AsGSTe1*, *AsGSTe2*, pseudogene *ψAsGSTe2, AsGSTe4* and *AsGSTe5*) was attempted, it was successful only in Che-S and Del strains, but no amplification took place in Alw-R strain. Assuming that the amplification may have failed in the Alw-R strain due to mismatch/es in the primer region, we tried alternative primer combinations, but no amplification was observed. Failure of PCR amplification of a genomic region from *AsGSTe1* through *AsGSTe5* in Alw-R was suspected to be due to structural variation (SV) in the genome, possibly, gene duplication, which may have displaced primer annealing sites too far apart to be amplified by an L-PCR (the maximum length we can amplify in our lab is ~ 12 kb). Successful amplification of genomic region encompassing AsGSTe2 through AsGSTe4 in Alw-R, but the failure of amplification of extended genomic region indicated the probable presence of SV breakpoints in the extended regions (*AsGSTe1* and *AsGSTe5*).

The PCR amplified products from Che-S and Del were sequenced which revealed an arrangement of GSTe genes as reported by Ayres et al.^25^. The annotated sequences have been deposited to GenBank (accession nos.: MZ052229- MZ052230).

**Figure 1:**
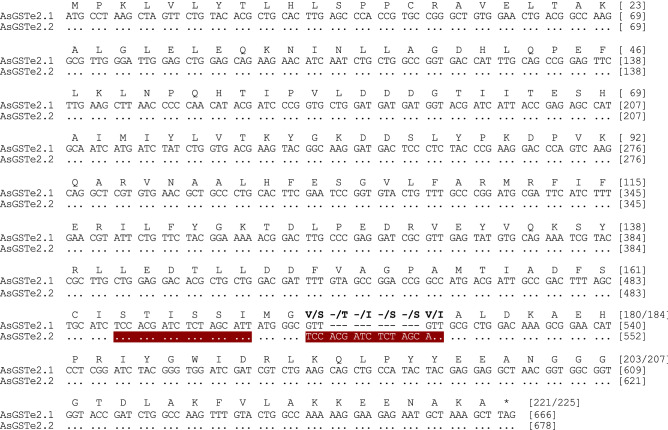
Alignment of nucleotide sequence (CDS) and deduced amino acids of full *AsGSTe2* paralogs present in all Alw-R mosquitoes. The two paralogs differed by 12 bp indel and four bp substitutions leading to the insertion of four amino acids in *AsGSTe2.2* and two amino acids substitutions. The new paralogs formed due to a repeat of the 18 bp motif (highlighted).

**Figure 2:**
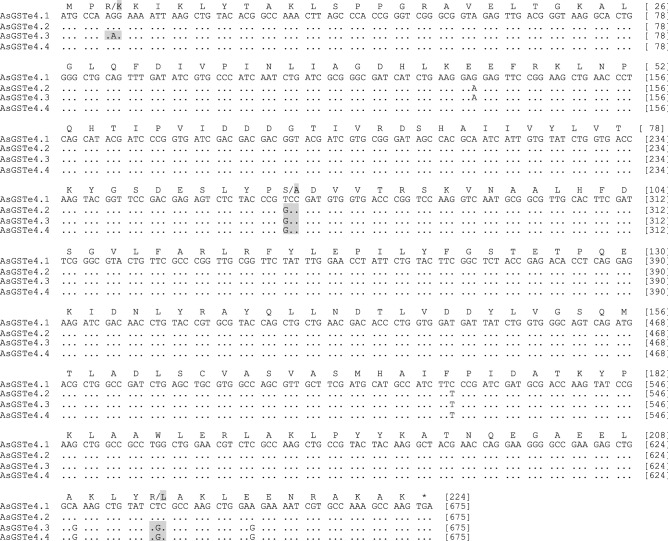
Alignment of nucleotide sequences and deduced amino acid sequences of *AsGSTe4* paralogs: *AsGSTe4.1* (Alw-R and Delhi strain); *AsGSTe4.2* (Alw-R); *AsGSTe4.3* (Alw-R); *AsGSTe4.4* (Che-S and SDA500).

**Figure 3:**
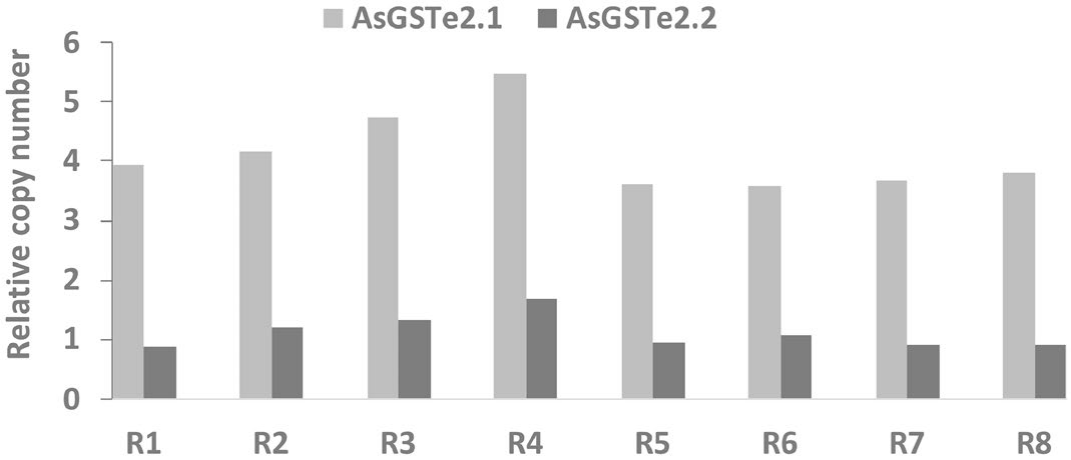
Disproportionate copy number of two GSTe2 variants (*AsGSTe2*.1 and *AsGSTe2*.2) in insecticide-resistant *An. stephensi*. The bar chart show the relative copy number of *AsGSTe2.1* and *AsGSTe2.1* with reference to endogenous genes in individual DDT-resistant line (Alw-R) mosquitoes (R1-R8).

For molecular characterization of GSTe genes in the Alw-R, the full coding regions of *AsGSTe2,* and *AsGSTe4* were sequenced using primers designed from the UTR regions. During sequencing of *AsGSTe2* from the gDNA of Alw-R (~ 940 bp)*,* we consistently observed extremely low-intensity secondary peaks in the DNA sequence chromatogram starting at a fixed nucleotide position in all samples (n = 15) from nucleotide position c.514 (in forward direction sequences; Supplementary Figure S1). Changing of primers did not alter the observation which ruled out asymmetric PCR amplification. Careful examination of low peak signals revealed the possible presence of an additional low copy variant having 12 bp indel/substitutions. On the other hand, direct sequencing of PCR product (~ 800 bp) targeting complete *AsGSTe4* from gDNA revealed heterozygosity at fixed nucleotide loci in all (n = 18) samples. Complete heterozygosity in both genes indicate the presence of duplicated genes involving *AsGSTe2* and *AsGSTe4*. Phasing out of haplotypes by sequencing cloned gDNA as well as cDNA from an individual mosquito confirmed the presence of two different variants of *AsGSTe2* and three variants of *AsGSTe4* present in a single individual. The aligned nucleotide and deduced amino acid sequences of the full coding region have been shown in Figs. 1 and 2, respectively. The two variants of *AsGSTe2*, designated as *AsGSTe2.1* and *AsGSTe2*.2, exhibited both length and nucleotide polymorphism. The differences in the two variants are derived from the insertion of 16 bp repeat (copied from base positions 490–507 and pasted at position 514) together with a deletion of four consecutive nucleotides (positions 514–517; highlighted in Fig. 1), which resulted in the addition of six amino acids and deletion of two amino acids in variant *AsGSTe2.2*. The proportion of *AsGSTe2*.2 clones derived from gDNA was relatively too small (three out of 21) as compared to *AsGSTe2.1* (18 out of 21). Variant-specific quantitative real-time PCR (qPCR) on the gDNA further confirmed a low copy number of *AsGSTe2.2* (with respect to the endogenous gene) as compared to *AsGSTe2.1* in different individuals (Fig. 3). The disproportionate numbers of two variants in gDNA are indicative of the presence of multiple copies of *AsGSTe2*.*1*, if we assume the presence of at least one copy of *AsGST2*.2 in the genome. Sequencing of 10 *AsGSTe4*-clones from gDNA as well as from cDNA, each derived from a single mosquito, revealed the presence of three variants in an individual mosquito, which were designated as *AsGSTe4.1*, *AsGSTe4.2* and *AsGSTe4.3* (Fig. 2).

The molecular characterization of pseudogene *ψAsGSTe2* (designated by Aiyer et al.^25^) was performed by cloning of PCR product because direct sequencing failed in all samples (n = 17) due to indels. A genomic region comprising pseudogene *ψAsGSTe2* and flanking regions of *AsGSTe2* and *AsGSTe4* (n = 20) derived from a single mosquito was cloned ans sequenced, which revealed the presence of two variants of the pseudogenes, designated as *ψAsGSTe2.1* and *ψAsGSTe2.2* (Figure S2). These two pseudogenes together with two variants of *AsGSTe2* form three haplotypes designated as Hap1 (*AsGSTe2.1* + *ψAsGSTe2.2*), Hap2 (*AsGSTe2.1* + *ψAsGSTe2.1*) and Hap3 (*AsGSTe2.2* + *ψAsGSTe2.2*). The proportion of clones representing Hap3 (linked to *AsGSTe2.2*) was considerably low (2/20) as compared to Hap1 (8/20) and Hap2 (10/20). This further confirmed the presence of a low copy number of *AsGSTe2.2*.

These observations indicated duplication of the GSTe gene array involving at the very least, *AsGSTe2, ψAsGSTe2* and *AsGSTe4* genes.

### Identification of duplication breakpoints

**Figure 4:**
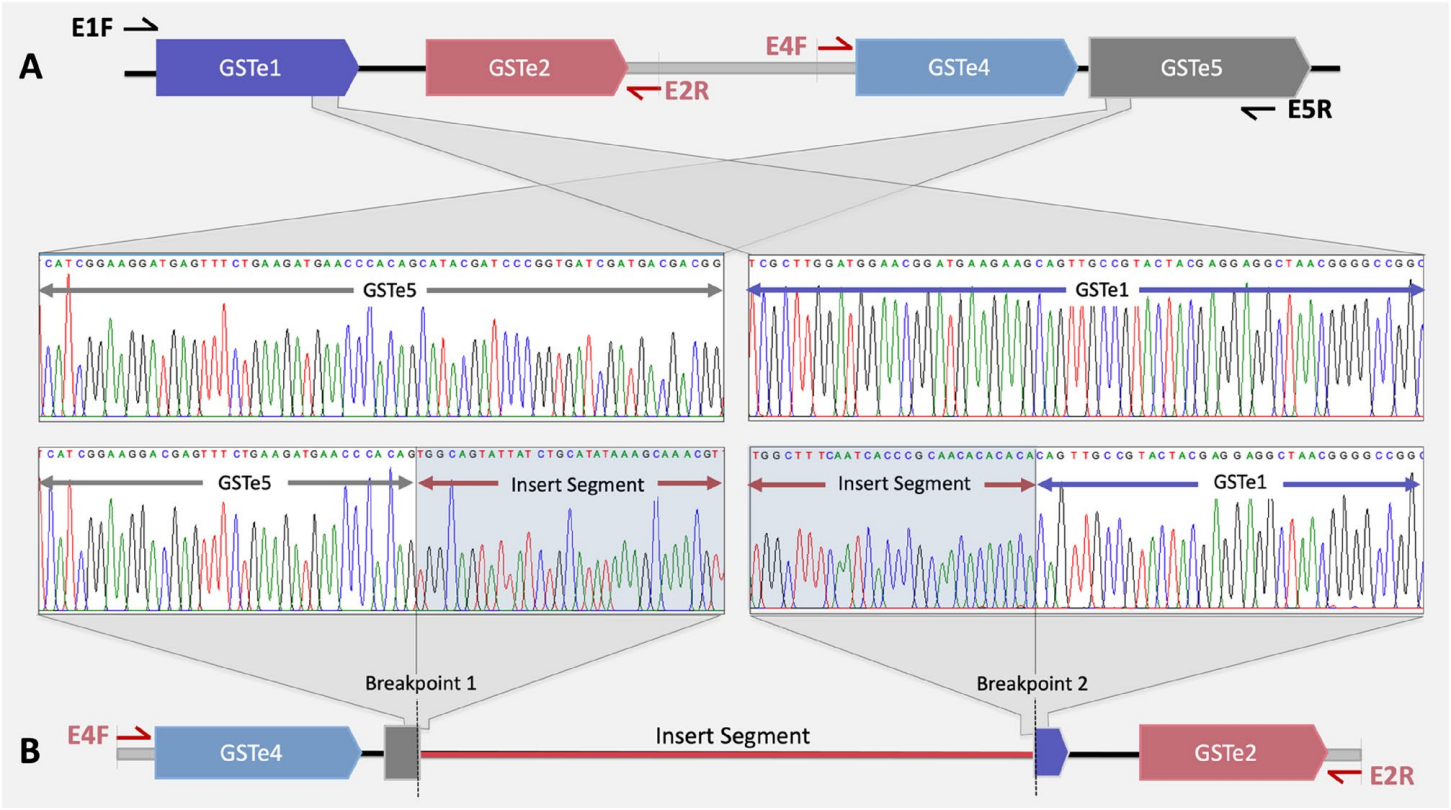
Identification of duplication-breakpoints through inverted primerPCR (**A**) Genomic arrangement of GSTe genes in the susceptible mosquito (Che-S strain) as revealed through sequencing of L-PCR product amplified from primers E1F and E5R (shown with black harpoons). No amplification was found in Alw-R with these primers. (**B**) Identification of duplication breakpoints in resistant mosquitoes as revealed through sequencing of PCR product amplified from inverted primers E2R and E4F (shown with red harpoons). Inverted primer PCR amplification was successful only in the Alw-R mosquito. Part of the DNA sequence chromatograms displayed here is from the genomic region flanking duplication breakpoints in resistant mosquitoes and the corresponding region in the susceptible mosquito. The shaded part of the chromatogram is the insert DNA segment.

To identify breakpoints and orientation of gene duplication, we attempted ‘Single-Primer PCR’ and ‘Inverted primer PCR’. Inverted primer PCR with primers designed from downstream of *AsGSTe2* (E2R) and upstream of *AsGSTe4* (E4F) was successful with Alw-R but not with Che-S and Del. The single-primer-PCRs failed. Successful PCR amplification with inverted primer PCR and failure of amplification with single primer PCR on all the samples of Alw-R (n = 20) indicates that the entire Alw-R colony has gene duplication in the same orientation (direct tandem duplication). The failure of inverted primer PCR and single-primer-PCR in Che-S and Del indicates the absence of gene duplication in the targeted genomic region. The amplified product was treated with Exo-Sap and sequenced through primer-walking. The genomic arrangement of the amplified product in Alw-R as revealed through sequencing has been displayed in Fig. 4. Analysis of sequences revealed the presence of two breakpoints, one in *AsGSTe5* and another in *AsGSTe1*, which were joined with a 2,423 bp insert segment (IS) (Fig. 4). The IS is unrelated to the genomic region representing GSTe cluster. The BLAST search revealed that a major portion of the IS (2374 bp ) is homologous to a genomic region located on a different chromosome, 2L (GenBank accession no: CP032233.1), while the GSTe gene cluster is located on chromosome 3R.

### Organization of GSTe gene array and duplication pattern

**Figure 5:**
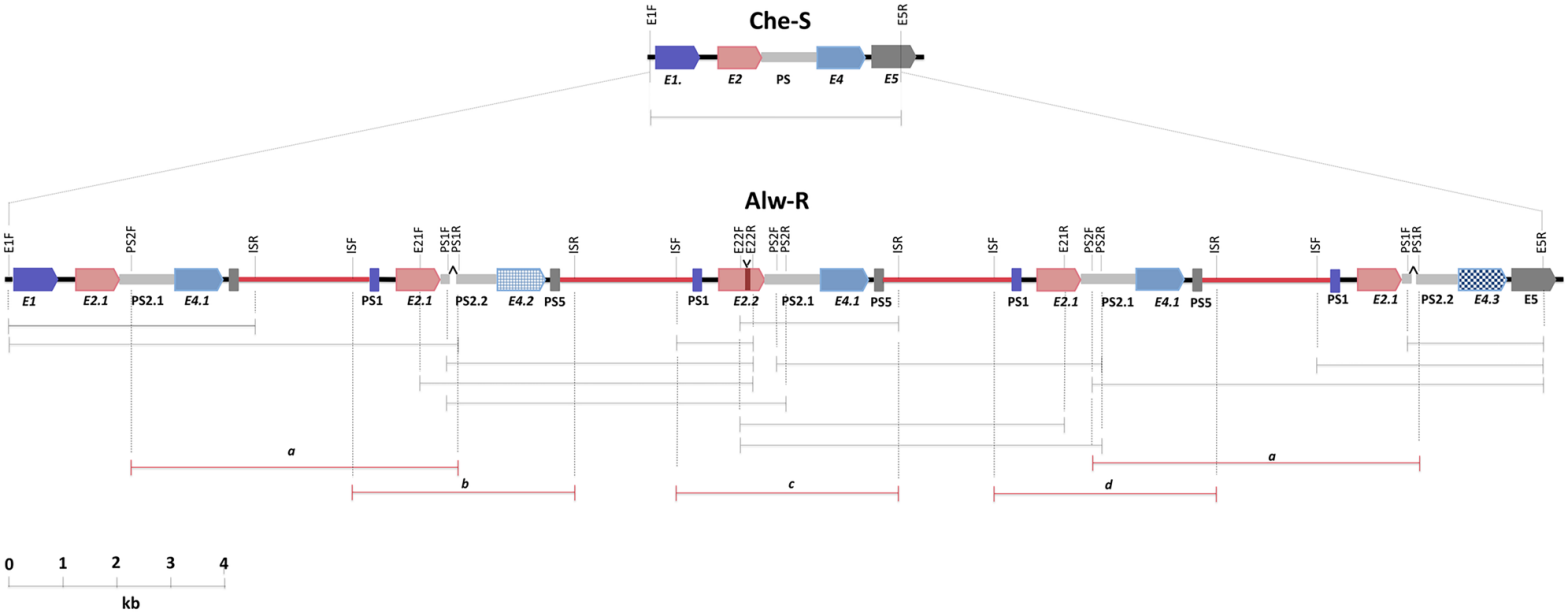
Schematic illustration showing arrangements of GSTe gene array in DDT-susceptible (Che-S) and -resistant (Alw-R) strains of *An. stephensi.* The arrangement of duplicated GST epsilon array in Alw-R (near-isogenic line) is based on the manual alignment of sequences derived from a series of L-PCR. The coloured blocks represent different GSTe genes. The black, red and grey bars connecting GSTe genes represent intergenic regions, insert-segment and *ψAsGSTe2*, respectively. The name and location of primers used for PCR amplification are shown in vertical text. Some primers, viz., E21F, E21R, E22F, E22R, PS1F, PS1R, PS2F, and PS2R are haplotype-specific and were designed from indel sites present in *AsGSTe2* and *ψAsGSTe2* pseudogene variants. Black horizontal dimension lines indicate the size of a successful PCR amplicon derived from primer sets. Red horizontal dimension-lines ‘a’ represents a PCR amplicon amplified by a single PCR that aligns to two regions. Red horizontal dimension lines, ‘b’, ‘c’ and ‘d’, represent a single amplicon having three haplotypes, which were phased out by sequencing using different haplotype-specific primers. The location of deletion in *ψAsGSTe2* and insertion in *AsGSTe2* have been indicated by the caret and upside-down caret symbols, respectively. Abbreviations used: E1: *AsGSTe1*; E2: *AsGSTe2;* E2.1: *AsGSTe2.1*; E2.2: *AsGSTe2.2*; E4.1: *AsGSTe4.1*; E4.2: *AsGST4.2*; E4.3: *AsGSTe4.3*; E5: *AsGSTe5*; PS2.1: *ψAsGSTe2.1*; PS2.2: *ψAsGSTe2.2*; PS1: truncated *AsGSTe1* (pseudogene); PS5: truncated *AsGSTe5* (pseudogene).

To explore the exact span of the genomic region being duplicated and the genomic organization of duplicated GSTe genes, PCR strategies were designed. The presence of long indels in *AsGSTe2* (12 bp) and *ψAsGSTe2* (23 bp) was exploited for designing indel-specific primers (E21F, E21R, E22F, E22R, PS1F, PS1R, PS2F, PS2R; Fig. 5) which were used for L-PCR amplification of gDNA and sequencing in combination with some other gene-specific primers. Indel-specific primers helped in amplification of each duplicated segment and annotation of sequences derived. The locations of all primers used for amplification and size of PCR-amplicons successfully amplified with specific primer-pair sets have been shown in Fig. 5. The amplicons were sequenced using the primer-walking strategy. The manually annotated sequence organization of the duplication region is also displayed in Fig. 5. The figure displays the most plausible but minimum number of duplications. It may be noted that in the case of the presence of two consecutive identical tandem duplications in the same direction, PCR will tend to amplify the shortest fragment; thereby we may miss some duplication events. It was observed that at least five tandem repeats of a 3.62 kb long units of GST epsilon array exist, each duplicated segment intercepted by a conserved IS. Each duplicated segment comprised of an array of partial *AsGSTe1*, *AsGSTe2*, *ψAsGSTe2*, *AsGSTe4* and partial *AsGSTe5* interconnected by an IS. Careful examination of the gDNA sequence revealed that the IS is non-coding and doesn’t appear to be a DNA-transposon in the absence of common transposon motifs such as terminal invert repeats (TIR), long terminal repeats (LTR), or target site duplication (TSD). All the duplicated segments were in the same direction and we failed to recognize any inverted gene duplication as attempts to amplify PCR using the same direction primers (forward-forward or reverse-reverse) failed. Attempts to amplify larger PCR products, more than 12 kb, also failed. During sequencing, all the two *AsGSTe2* and three *AsGSTe4* variants were identified in the duplicated arrangement, that was explored after sequencing of cloned product earlier. Interestingly, we did not find polymorphism in IS repeats. The breakpoints of all duplicated events are identical, one in the *AsGSTe1* gene and the other in *AsGSTe5* resulting in the incorporation of truncated *AsGSTe1* and *AsGSTe5* in duplicated segments.

### Confirmation of gene duplication through whole-genome sequencing in Alw-R

**Figure 6:**
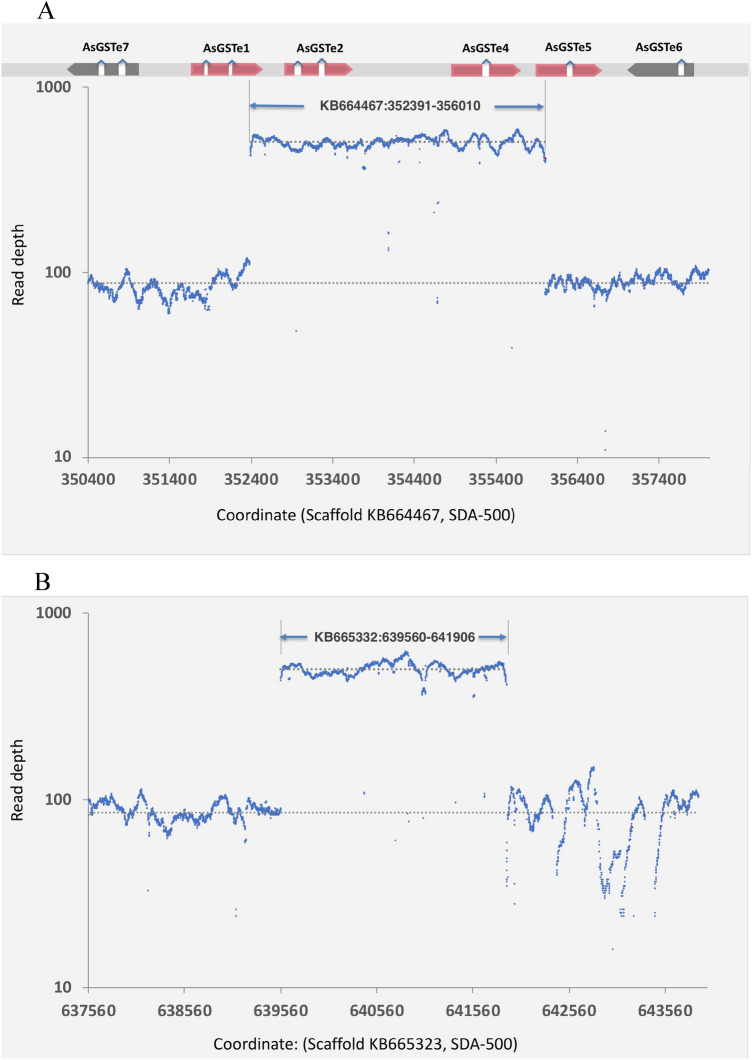
Coverage plot of Illumina sequence reads aligned against the reference genome (SDA500) showing duplicate region (with high read-depth) along with flanking 2 kb region. A: GSTe gene cluster (Scaffold: KB664467. B: insert segment (IS) on a different chromosome (Scaffold: KB665323). A bar on the top of figure ‘A’ depicts the location of various GSTe genes on the scaffold. The dotted grey line is the mean read depth of the duplicated and flanking regions.

For the confirmation of the duplication event, Ilumina paired-end whole genome sequence of Alw-R was obtained and subjected to structural variation (SV) analysis using the tool LUMPY^26^. The analysis did not provide any significant duplication (DEL) or inversion (INV) of any full-length gene. To confirm domestic insertion (DIN) of IS (mapping on different chromosomes) at the duplication breakpoints in the GSTe cluster, as demonstrated through Sanger’s sequencing, we checked for break ends (BND)-SV. Two pairs of BNDs were recorded with mates located on scaffolds KB664467 and KB665332 (SDA500, VectorBase). The breakpoints were identified on scaffold KB664467 (bearing GSTe cluster) at coordinates 352391 and 356010, which are identical to what we demonstrated through Sanger sequencing of L-PCR products. However, breakpoints identified on scaffold KB665332 (at coordinates 639560 and 641906) corresponding to IS is shorter by 49 bp at one end than the actual size of IS as determined through Sanger’s sequencing. This is because of the absence of a matching 49 bp sequence in the reference genome. To verify the presence of gene-duplication, read-depth coverage data of a region between identified breakpoints were compared with the flanking 4 kb regions (2 kb each side) which are free from duplication or deletion events (Fig. 6). The ratio of mean read depth of duplicated regions with flanking regions was found to be 5.8 and 5.9 (log2-fold: 2.5 and 2.6), respectively, for GSTe cluster and IS; indicating occurrences of duplication ~ 5 to 6 times.

### Enhanced mRNA transcript number associated with duplication

**Figure 7:**
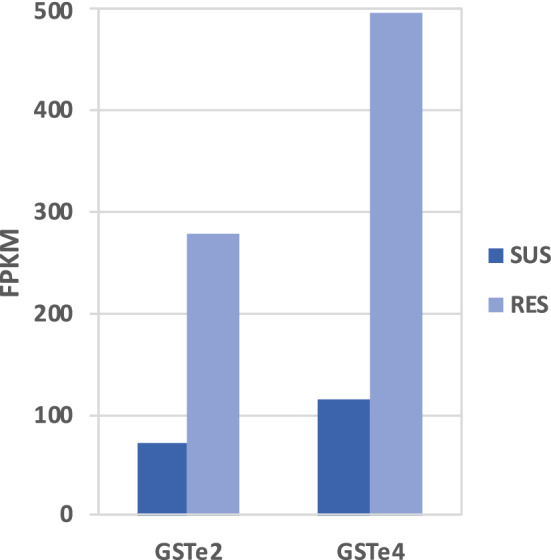
Fragments per kilobase of transcript per million fragments mapped (FPKM) for AsGSTe2 and AsGSTe4 in DDT-susceptible (Che-S) and -resistant (Alw-R) *An. stephensi*.

Differential gene expression analysis of RNAseq data revealed significant upregulation of a total of 97 genes (NOISeq q = 0.95; GFOLD > 1) in Alw-R as compared to Che-S, which includes *AsGSTe2* (ASTE016034) and *AsGSTe4* (ASTE016035) (Supplementary Table S2 and S3). The normalized FPKM values for *AsGSTe2* and *AsGSTe4* in Alw-R and Che-S have been displayed in Fig. 7.

### Phylogenetic analysis of *ψAsGSTe2*

**Figure 8:**
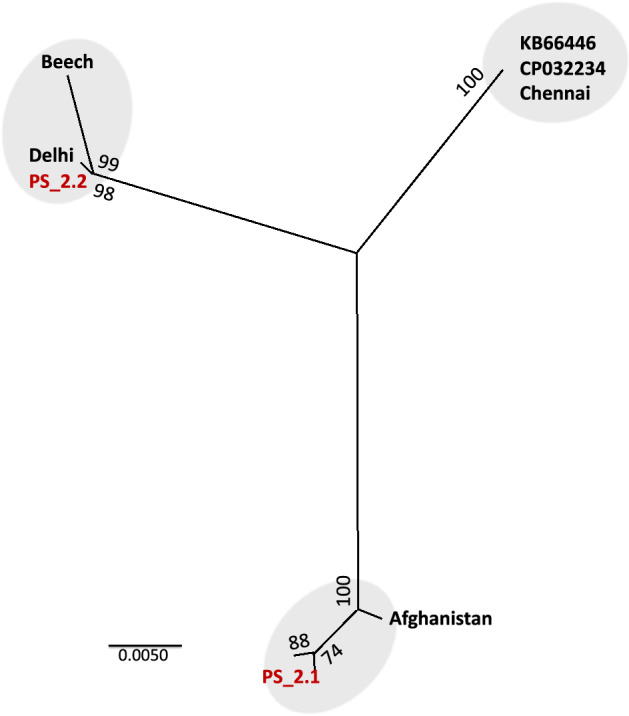
The maximum-likelihood phylogenetic tree inferred from all known *ψAsGSTe2* depicting three distinct clades. PS2.1 and PS2.2 are two *ψAsGSTe2* variants incorporated in a duplicated gene array in Alw-R. KB66446 and CP032234 represent scaffolds from two WGS assemblies (SDA-500, originating Pakistan) from where *ψAsGSTe2* sequences were downloaded. Delhi and Chennai represent the locality of strains having a single copy of *AsGSTe*. Sequences of Beech (origin: Delhi) and Afghanistan were sourced from Ayres et al.^25^. Numbers shown on nodes indicate bootstrap value.

It has been reported that *ψAsGSTe2* is transcribed and conserved, and therefore may have a role in regulatory function^25^. We performed a phylogenetic analysis of two variants of *ψAsGSTe2* found in this study along with earlier reported sequences and sequences retrieved from WGS databases. The best model for phylogeny as determined by MEGA-11^36^ was T92+G, based on the lowest Bayesian Information Criterion (BIC) scores. The maximum-likelihood tree constructed using Tamura 3-parameter (T29) with 500 bootstraps, shows three distinct clades of *ψAsGSTe2* (Fig. 8). The *ψAsGSTe2.1* from Alw-R is nested in a clade with Afghanistan, *ψAsGSTe2.2* with Beech strains. The third clade consisted of Che-S strain and two WGS databases (scaffolds KB66446 and CP032234), which were 100% identical.

## Discussion

Gene amplification and duplication have often been reported associated with insecticide resistance^37,38^ and may either increase the number of transcripts of insecticide metabolizing genes or act to partially compensate the fitness cost associated with mutant alleles conferring insecticide resistance^38^. In general, gene duplication serves as a major driving force in the evolution of new genes, which may play important roles in the adaptation to changing environments^35^ through neofunctionization or subfunctionization. In this study, we report the tandem duplication of a genomic region encoding two insecticide resistance-associated enzymes, GSTe2 and GSTe4. Glutathione S-transferases play important roles in resistance to DDT and other groups of insecticides. One of the major known mechanisms of DDT resistance in mosquitoes is the overexpression of the enzyme GSTe2^39^ or non-synonymous changes in the gene sequence^28^ which result in enhanced metabolic activity. In this study, we report an increased copy number of two GST epsilon genes, *AsGSTe2* and *AsGSTe4*, in DDT-resistant *An. stephensi* due to tandem duplication that is translated into the increased number of mRNA transcripts (Fig. 7). The gene duplication event in this strain also likely facilitated the diversification of GSTe2 and GSTe4 genes with the presence of two and three paralogs, respectively, in an individual. The two paralogs of *AsGSTe2* differ by six consecutive amino acids, due to the substitution of two amino acids and insertion of four amino acids. The genomic organization study, as well as qPCR data on gDNA, suggests that *AsGSTe2*.2 is present in a single copy, while *AsGSTe2.1* is present in multiple copies. All the paralogs of these two genes seem to be functional with no frameshift or premature stop codon resulting from mutations and transcribed into mRNA. Whether all paralogs of both *AsGSTe2* and *AsGSTe4* confer resistance to DDT is not known, but it has been shown that recombinant enzymes of two paralogs of *AsGSTe2* genes can metabolize DDT^40^. The possible role of *AsGSTe4* paralogs in insecticide resistance is not known yet. However, the retention of three different paralogs of *AsGSTe4* in a duplicated GST array may have some positive functional role.

The mechanism of the initial gene duplication event, in this case, is not clear. There are a number of proposed mechanisms of gene duplication and amplification^41^, however, none of the models explains the mechanism of duplication in the present case. Transposable elements are known to contribute to gene duplication through transposition and by serving as portable regions of homology that support homologous recombination. Recent whole genome sequencing of *An. stephensi* has shown insertions of transposon in functional genetic elements including the CYP450 gene array^42^. However, the role of the transposon is ruled out in this case due to the absence of common transposon-motifs such as terminal invert repeats (TIR), long terminal repeats (LTR), or target site duplication (TSD). Interestingly, the duplication event is associated with the insertion of 2.4 kb of IS connecting two duplicated segments of the GSTe array. A major portion (98%) of the IS is homologous to a genomic region that maps to a different chromosome (2L), while the GSTe array is mapped to chromosome 3R. In an earlier study, Ayres et al.^25^ found that *ψAsGSTe2* pseudogene is transcriptionally active and suggested that it may have a regulatory function. Though pseudogenes lack regulatory elements, they may alter gene expression by acting as small interfering RNA or competing for endogenous mRNAs as reported elsewhere^43^. Our study shows the presence of at least three diverged clades of pseudogene (Fig. 8) but with almost no variation within each clade (Figure S2). The three haplotypes of the pseudogene, two retrieved from WGS data (SDA-500) and one we found in Chennai strain, are highly conserved without a single base change, despite the fact that the SDA500 strain (originating Pakistan) and Chennai (Southern India) strains are from geographically distant localities. Similar conservation in pseudogene is also noted between Delhi strain, Beech strain (originating Delhi) and variant *ψAsGSTe2.2* of Alw_R, and between variant *ψAsGSTe2.1* of Alw-R and Afghanistan strain. Such conservation in *ψAsGSTe2* pseudogene sequences, suggests that they are under selective constraint, possibly due to a regulatory function. The co-occurence of two highly diverged paralogs of *ψAsGSTe2* in Alw-R, which are already present in different populations, indicates that the inclusion of two diverged paralogs of *ψAsGSTe2* in Alw-R is most likely through recombination of two diverged strains, not through sequential evolution of duplicated *ψAsGSTe2*.

Most of the studies on insecticide resistance are based on over-expression at the mRNA level, which fails to recognize copy number variation at the genomic level. The present study is an example where overexpression of insecticide detoxifying genes is due to gene duplication. Though gene duplication may be less frequently observed than single nucleotide mutations^44^, they appear to be a common adaptive response to varying environments. Whole genome sequencing of field mosquitoes resistant to insecticides may unravel copy number variation in insecticide metabolizing genes. Lucas et al.^44^ identified as many as 28 genes containing CNV form families linked to metabolic insecticide resistance. The possible role of transposons in *An. stephensi*^42^ and inversion in chromosome^55^ in gene duplication and insecticide resistance also need to be investigated. Thakre et al.^55^ reported the presence of some GST and CYP450 genes in the chromosomal inversion 2R*b* region; however, GSTe genes are present on a different chromosome.

This study provides de-novo evidence of duplication and elevation of gene dosage of two *AsGSTe* genes in DDT-resistant *An. stephensi.* However, the functional role of increased doses of *AsGSTe2* and *AsGSTe4* in insecticide resistance, frequency and distribution of population with such gene duplication in field population is required to be investigated. The resistant strain of *An. stephensi* used in this study is fixed for L1014S mutation and we could not colonize and maintain other strains with different *kdr* genotypes.

## Material and methods

### Mosquito samples

A DDT-resistant line of *An. stephensi* (LT_50_: 14 h with 4% DDT) collected from Alwar district, Rajasthan (27°33′ N 76°37′ E) in year 2012 was selected in the laboratory by exposing adult female mosquitoes to a sublethal dose of DDT in intermittent generations with an increasing dose determined empirically. Mosquitoes collected from Chennai (13°04′ N 80°12′ E) in the year 2015 with a low level of resistance against DDT (69% mortality with 4% DDT) were screened for susceptible individuals by exposing them to WHO’s 4% DDT-impregnated paper. Mosquitoes knocked down within 10 min were used in this study as susceptible mosquitoes. Genomic DNA of an old *An. stephensi* preserved from the insectary maintained in the National Institute of Malaria Research’s insectary (originating from Delhi with unknown insecticide susceptibility status) since the 1990s, were also used for DNA sequencing of GSTe genes. Genotyping of these strain for *kdr* alleles using method by Singh et al.^11^ revealed that the Alw-R line was homozygous for the L1014S *kdr-*allele while other mosquitoes used in this study (Che-S and Delhi) were with wild type *kdr* allele.

### Molecular characterization GSTe genes

#### Long PCR amplification and sequencing of GSTe gene array

The genomic DNA of *An. stephensi* was isolated using the method by Black and Duteau^45^. The gDNA was subjected to L-PCR using different primer pair sets: (i) E2F (forward) and E4R (reverse) designed from 5 UTR of *AsGSTe2* and 3 UTR of *AsGSTe2*, respectively, and, (ii) a forward primer (E1F or E1F2) designed from *AsGSTe1* and a reverse primer (E5R or E5F2) designed from *AsGSTe5*. The PCR reaction contained 1X LongAmp Taq reaction buffer (New England BioLabs), 2.5 units of LongAmp Taq DNA polymerase, 300 µM dNTPs, 0.2 µM of each primer and 0.5 µl of gDNA in a total reaction volume of 25 µl. The thermal cycling conditions were: an initial denaturation step at 94 °C for 3 min, followed by a touchdown step of 20 cycles, each with denaturation step at 94 °C for 30 s, annealing at temperature starting from 65 °C with an increment of − 0.5 °C per cycle for 30 s and an extension at 72 °C for 5 min; followed by 15 cycles each with denaturation step at 94 °C for 30 s, annealing at 55 °C and extension at 72 °C for 5 min. A final extension step was performed at 72 °C for 15 min. In the case of the Alw-R mosquito, due to PCR failure, the extension time was increased up to 15 min. The successful PCR product (Delhi and Che-S only) were sequenced through the Sanger method using the primer-walking strategy. Briefly, the PCR products were cleaned using ExoSAP-IT (Thermo Fisher Scientific), sequence termination reaction was performed using BigDye Terminator v3.2 (Invitrogen Inc) and the cleaned sequence termination products were electrophoresed in ABI Prism 3730xl.

#### Molecular characterization of *AsGSTe2*, *AsGSTe4* and *ψAsGSTe2* pseudogenes in Alw-R

For molecular characteeization of GSTe genes in Alw-R, gDNA as well as cDNA isolated from individual mosquitoes were used. For cDNA synthesis, total RNA was isolated from individual mosquito and cDNA was synthesized using GoScript Reverse Transcription System kit (Promega Corporation). For molecular characterization of *AsGSTe2* and *AsGSTe4*, both the gDNA and cDNA were PCR-amplified, and for *ψAsGSTe2* only gDNA was amplified. PCR amplification was carried out in 20 µl reaction containing 0.5 units of AmpliTaq Gold (Invitrogen Inc) 200 µM of dNTPs, 0.2 µM of each primer and 0.5 µl of gDNA or 2 µl of cDNA. Primers used for amplification of *AsGSTe2* were E2F and E2R, and for *AsGSTe4* were E4F and E4R (Supplementary Table S1). The *ψAsGSTe2* and flanking region was PCR-amplified using primers E2qF (designed from GSTe2) and PSR (designed from GSTe4) (Supplementary Table S1). The PCR conditions were an initial denaturation at 95 °C for 3 min, followed by 35 cycles, each comprising of denaturation at 95 °C for 30 s, annealing at 55 °C for 60 s, denaturation at 72 °C for 1 min, and final extension at 72 °C for 7 min. The PCR products were sequenced as described above.

For cloning, gDNA as well as cDNA of *AsGSTe2, AsGSTe4* and gDNA of *ψAsGSTe2* were amplified with Phusion High-Fidelity PCR Master Mix with HF Buffer (New England Biolabs) using 0.1 µM of each primer as described above. Two-step PCRs were carried out with common PCR conditions, i.e., an initial denaturation at 98 °C for 30 s followed by 35 cycles each with denaturation step at 98 °C for 10 s and annealing/extension step at 68 °C for 2 min, and a final extension at 72 °C for 10 min. For cloning, the amplified products were purified using QIAquick PCR Purification kit (Qiagen Inc) and incubated at 72 °C for 10 min in a reaction mixture (25 µL) containing 200 µM of dATP, 1.5 mM MgCl_2_, 0.625 unit of Taq DNA polymerase and 1X buffer, to add A-tail. Cloning and sequencing were performed following Mishra et al.^46^.

### Quantitative PCR

Quantitative PCR (qPCR) in triplicate were performed, each in 10 μl of reaction mixture containing 0.3 µM of each primer, 1X SYBR Green Realtime PCR master mix (Toboyo Co., Ltd, Japan) and 0.5 µl of gDNA template. qPCR cycling conditions were: initial denaturation at 94 °C for 5 min, followed 40 cycles each with denaturation at 94 °C for 15 s, annealing at 60 °C for 20 s and extension at 72 °C for 30 s, followed by dissociation curve analysis. Primers used for variant *AsGSTe2.1* were E21qF and E21qR, for variant *AsGSTe2.2* were E22qF and E22qR, and for common *AsGSTe2* were E2qF and E2qR. Primers St_S7F and St_S7R targeting ribosomal protein S7 (endogenous gene) were used for the normalization of data. PCR-efficiencies of all primer-sets were determined by running qPCR on a series of DNA templates serially diluted by 1/10^th^ factor.

### Identification of breakpoints and orientation of duplication

**Figure 9:**
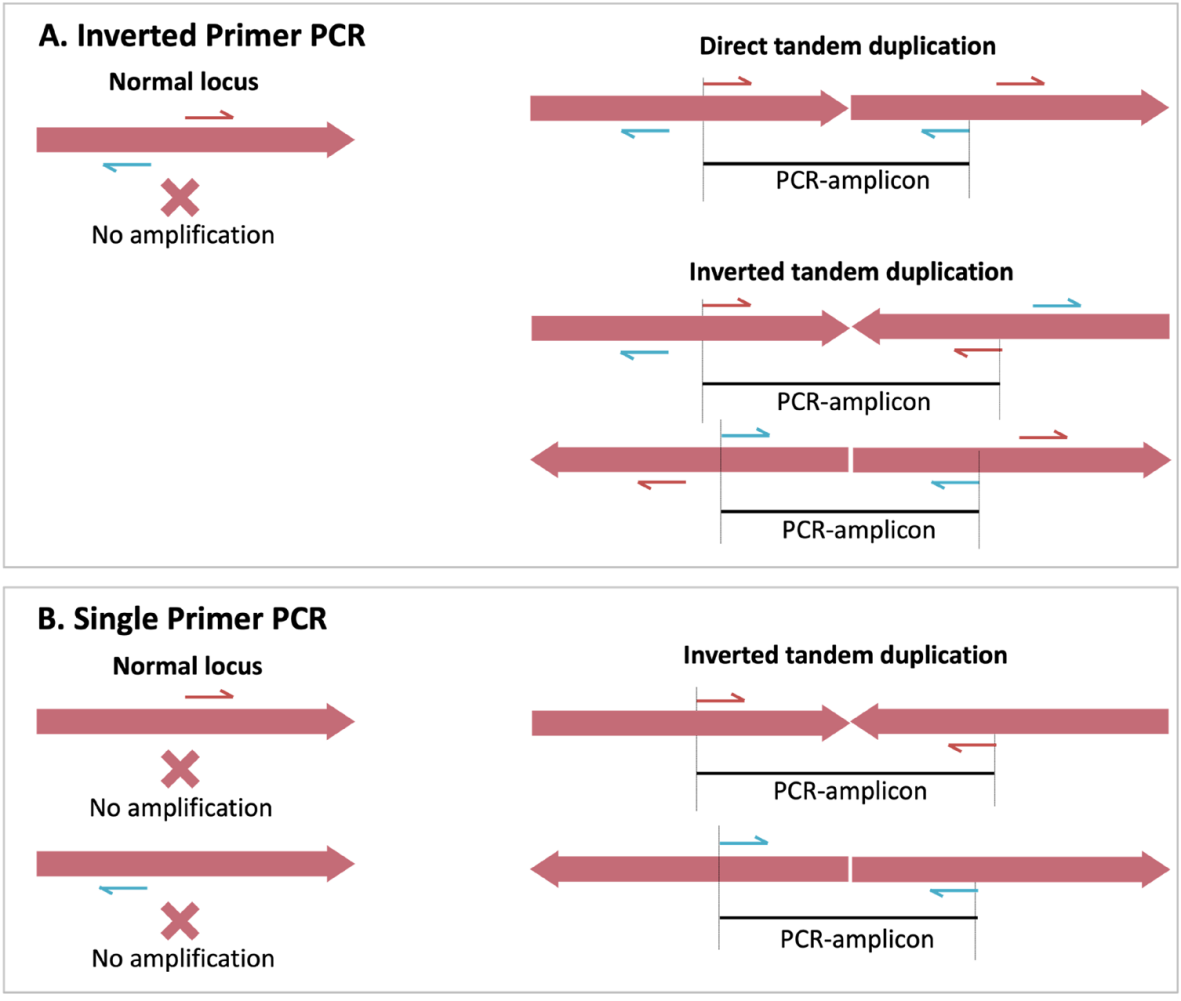
PCR strategies to detect tandem gene duplication. (**A**) PCR with inverted (outward facing) primer pair. Successful PCR-amplification indicates presence of direct or inverted tandem duplication. (**B**) PCR with single primer (Single Primer PCR): Successful PCR amplification indicates presence of inverted tandem duplication. Harpoon represents primers, solid red arrow bar represents a genomic locus and black horizontal line represents PCR amplicon.

To identify breakpoint and orientation of duplication, we performed modified ‘inverse-PCR’^47^ as well as ‘single-primer PCR’ on gDNA. In classic ‘inverse-PCR’, target DNA is circularized through ligation of restriction-digested DNA, followed by PCR-amplification using outward facing primer pair (inverted). In modified procedure, we used inverted primer directly on gDNA for PCR amplification without restriction digestion and ligation (referred herein ‘inverted primer PCR’. The diagramic representation of ‘inverted primer PCR’ and ‘single primer’ is shown in Fig. 9. The PCR with inverted-primer will produce amplicon only when direct or inverted tandem duplication is present, whereas, the single primer PCR amplification will produce amplicon only when inverted tandem duplication is present. Inverted primer PCR was carried out on gDNA isolated form Alw-R, Che-S and Del using two primers, E2R and E4F (facing outward, see Fig. 4) designed from the downstream flanking region of GSTe2 and upstream flanking region of GSTe4, respectively. Single-primer PCR was carried out using either E2R or E4F. Both PCRs were carried out using the L-PCR protocol (described above). The PCR products were purified using ExoSAP-IT (Thermo Fisher Scientific) before sequencing. Sequencing was performed using the primer-walking strategy.

### Organization of GSTe gene array and duplication pattern in Alw-R strain.

It was observed that the inverted primer PCR product contained mixed haplotypes; therefore we designed variant-specific primers (both forward and reverse) based on two polymorphic loci, one in GSTe2 coding region (E21F, E21R, E22F and E22R) and another in e2-pseudogene (PS1F, PS1R, PS2F, PS2R) (Fig. 5). The presence of 12 bp indel in GSTe2 and 23 bp indel in *ψAsGSTe2* were exploited for designing haplotype-specific primers. It was also discovered through sequencing of PCR product (described above) that there is an insert segment (IS) intervening duplication breakpoint. Primers in opposite directions were also designed from IS. A series of L-PCR were performed on an individual from a near-isogenic line isolated from Alw-R strain using combinations of these primer-pairs along with other primers. The primers used for L-PCR have been shown in Fig. 5 and have been listed in Supplementary Table S1. All L-PCR were carried out as described in the earlier section, with the exception that varied extension time between 6 to 15 min was used. The diagrammatic representation of successful PCR amplification with specific primer pairs and relative amplicon sizes have been shown in Fig. 5.

### Phylogentic analyses

The alignment of DNA sequences and phylogenetic tree construction was performed using MEGA11^36^. The alignment was performed using MUSCLE and the best model for phylogenic inference was determined using lowest Bayesian Information Criterion (BIC) scores. The maximum-liklihood tree was constructed with 500 bootstrap replicates.

### Whole-genome sequencing (WGS) and SV analyses

For WGS, gDNA was isolated from a pool of six female *An. stephensi* individuals of Alw-R strain using DNeasy Blood & Tissue Kits (Qiagen Inc) including an RNase treatment according to manufacturer’s protocol. DNA integrity quantity check, library preparation and sequencing were performed at Macrogen Inc. (South Korea) using the Illumina platform. Pair-end libraries (151 bp) were constructed using Library Kit TruSeq Nano DNA Kit following protocol TruSeq Nano DNA Sample Preparation Guide, Part # 15041110 Rev. D. Sequencing was performed on Illumina HiSeq2500. A total of 27 Gb of raw sequence data (100X coverage) was generated. The quality control tasks were performed by Trimmomatic-0.38 software^48^ to remove the adapter sequences, low-quality bases and minimum read length. The framework, SpeedSeq^49^ was used to align paired-end FASTQ sequences with the reference sequence (SDA-500) by Burrows-Wheeler Aligner (BWA)^50^ as well as extraction of discordant and split reads in the form of BAM files through Samblaster tool^51^, followed by detection of structural variants (SVs) breakpoint by LUMPY^26^,). The manual inspection was carried out by Integrative Genomic Viewer (IGV) tool^52^ to reach optimum annotation of the breakpoint in the context of SV types. The position-wise read depth was calculated using SAMtools (http://www.htslib.org/).

### RNAseq and differential gene expression analyses

Three to four-days old non-blood fed *An. stephensi* from Alw-R line were exposed to WHO’s DDT 4% impregnated insecticide paper for eight hours and Che-S mosquitoes were exposed for 15 min. Post-exposure active mosquitoes from Alw-R line (resistant) and knock-downed mosquitoes from Che-S line (susceptible) were selected for RNA isolation. Total RNA was isolated from a pool of six female individuals from each resistant and susceptible group using TRIzol reagent (Invitrogen, CA, USA). RNA samples after a quality check (with RIN > 8) were processed for RNAseq library preparation at M/s Scigenom (India). Briefly, 100 bp paired-end Illumina libraries of mRNA were prepared using Trueseq RNA Library Prep kit and sequenced on Illumina HiSeq2000. The quality check and trimming of sequences were done as described earlier. Mapping of processed reads was carried out using HISAT2 (HIASAT2.1.0) splice aligner program. The featureCounts tool was used to quantify the gene expression of mapping reads to each gene with the aid of a gene transfer format (GTF) file that bears the structure of transcripts of those genes. The output of gene quantification was subjected to normalization. In the absence of replicates, the analysis was performed by two packages: NOIseq^53^ and GFOLD (generalized fold change)^54^ using R. NOIseq runs by non-parametric and GFOLD, by a posterior distribution of log fold change approach to calculate the expression level of the genes. The RPKM normalization was applied by these packages to get an accurate surmise at the expression level. The differentially expressed features were selected afterward by setting a higher threshold of q = 0.9 (q = probability of differential expression) and GFOLD (0.01) > 1 by NOIseq and GFOLD package, respectively.

### Ethics approval

Approval from the Institutional Animal Ethics Committee and Institutional Biosafety Committee was obtained for the colonization of mosquitoes and the use of rabbits for feeding mosquitoes. All experiments were performed in accordance with relevant guidelines and regulations, including ARRIVE guidelines.

## Supplementary Information

Supplementary Information.

## Data Availability

All relevant data are within the paper. DNA sequences have been submitted to the NCBI’s GenBank database (GenBank accession no. MZ052229-MZ052230).
