# Supplementary material for: Tandem duplication of a genomic region encoding glutathione S-transferase epsilon-2 and -4 genes in DDT-resistant Anopheles stephensi strain from India
Source: Sci Rep. 2022 Oct 25;12:17872. doi: 10.1038/s41598-022-21522-8 (PMC9596695; doi:10.1038/s41598-022-21522-8)
Supplement: Supplementary file 1 — Supplementary Information. [file 41598_2022_21522_MOESM1_ESM.pdf]

Tandem duplication of a genomic region encoding glutathione S transferase epsilon-2 and -4 genes in DDT-resistant *Anopheles stephensi* strain from India

Cherry L. Dykes†[1], Gunjan Sharma†[1], Abhisek K. Behera[1], Neera Kapoor[2], Mark J. I. Paine[3], Martin J. Donnelly[3], Om P. Singh*[1]

[1]National Institute of Malaria Research, Sector 8, Dwarka, New Delhi-110077, India
[2]Indira Gandhi National Open University, Maidangarhi, New Delhi-110068, India
[3]Department of Vector Biology, Liverpool School of Tropical Medicine, L3 5QA, United Kingdom

†Contributed equally

*Corresponding author (dr.opsingh@gmail.com; singh@nimr.org.in)

Supplementray information

Table S1 List of primers used in the study

Name of primer	Sequence (5′—3′)
1. E1F	TCA GTT CAT TGC CGA CTT TG
2. E5R	CCT GGT TGA GCT GCT CGT A
3. E1F2	TGA AGC CGG AAT TCT TGA AGG
4. E5R2	TAG CTT TGA TCG CAT TTT TAA GTC T
5. E2F	CTC CAA CGA CCA CAA TCA TG
6. E2R	TGC TTC AAG TTA CGT TTG TGC
7. E4F	CTT GCA AGC GAG TGG AAC T
8. E4R	ATC ACT TCG AAC AGA CGC TG
9. E2qF	GCC GAC TTT AGC TGC ATC TC
10. E2qR	TCT CTT CCT TTT TGG CCA GTA
11. E21qF	TCT AGC ATT ATG GGC GTT GTT G
12. E21qR	CTT TGT CCA GCG CAA CAA C
13. E22qF	CTA GCA TTA TGG GCT CCA CG
14. E22qR	CTT TGT CCA GCG CAA TGC T
15. St_S7F	TGC GTG AAT TGG AGA AGA AGT T
16. St_S7R	CAG GAT GGC ATC GTA CAC AG
17. PSR	CGA TCA GAT TGA TGG GCA CG
18. PS1F	GTC AGG CCA TCA CAG CTT G
19. PS1R	TTG ACC AGC TCC AAG CTG TG
20. PS2F	CAG ACC ATC ACA GGG CAG T
21. PS2R	CTG ACC AGC TCC AAC ACC AT
22. ISF	CCG AAT ACG GTA AGC TCC AA
23. ISR	CGT CGT GTG CTA AAC GAT GA

Table S2 List of upregulated genes based on NOISeq's analysis

('M' is the log2-ratio of the two conditions, and 'D' is the value of the difference between conditions)

Gene ID	Mean expression		Differential expression statistics		Probability	Ranking
	Alw-R	Che-S	M	D		
ASTE006325	992.0955207	94.50517752	3.392013757	897.5903431	0.978304979	897.5967524
ASTE006367	197.7640497	22.85166178	3.113409204	174.9123879	0.966520993	174.9400948
ASTE016435	2167.430819	536.1910046	2.015167024	1631.239814	0.954502887	1631.241059
ASTE016494	224.35614	61.60749918	1.864612788	162.7486408	0.953644451	162.7593219
ASTE016243	136.8400892	32.84926381	2.058558006	103.9908254	0.955595442	104.0111986
ASTE005881	126.4878898	34.73743078	1.864436306	91.75045906	0.953644451	91.76940046
ASTE005891	304.7952635	66.06163664	2.205955866	238.7336269	0.956453879	238.7438184
ASTE004102	86.11236273	19.09953511	2.17268285	67.01282762	0.956453879	67.04803962
ASTE010150	276.5655372	63.13255712	2.131165301	213.4329801	0.956453879	213.4436198
ASTE008971	99.58386888	3.025908604	5.040471748	96.55796028	0.996722335	96.6894309
ASTE002155	14.9921682	1.161948904	3.689590511	13.8302193	0.970110816	14.31391085
ASTE002156	1385.654558	72.52497742	4.255945888	1313.12958	0.987162479	1313.136477
ASTE009555	77.6512431	21.66550561	1.841609116	55.98573749	0.953410332	56.01601848
ASTE007704	55.40668664	3.679504863	3.912476549	51.72718178	0.983728734	51.87493429
ASTE001895	92.00005428	14.66960491	2.648804696	77.33044936	0.959380365	77.3758009
ASTE014827	26.86502962	3.074323142	3.127388709	23.79070648	0.962306852	23.99538029
ASTE011694	350.4931078	80.65861975	2.119485452	269.8344881	0.956453879	269.842812
ASTE010348	28.42468301	5.228770068	2.442600725	23.19591294	0.95395661	23.32416506
ASTE010406	765.3024223	156.1610912	2.292994926	609.141331	0.957468394	609.1456468
ASTE010010	57.12230537	4.647795616	3.619435672	52.47450975	0.983728734	52.59918714
ASTE008531	43.14391187	0.145243613	8.214538458	42.99866826	0.999258623	43.77629626
ASTE005696	292.0840884	52.43294429	2.477838302	239.6511441	0.958443889	239.6639534
ASTE006192	561.416733	141.007341	1.993301792	420.409392	0.954424848	420.4141175
ASTE010525	106.7387788	27.16055563	1.974499512	79.57822317	0.954385828	79.6027151
ASTE009456	942.1866122	147.9790344	2.670620043	794.2075778	0.959380365	794.212068
ASTE010606	24.21361886	4.454137465	2.442600725	19.7594814	0.952278758	19.90988205
ASTE004212	1112.266814	287.1224156	1.953765037	825.1443986	0.954346808	825.1467117
**ASTE016034 (AsGSTe2)**	**236.1510187**	**60.39713574**	**1.967157721**	**175.753883**	**0.954385828**	**175.7648915**
**ASTE016035 (AsGSTe4)**	**447.5230443**	**102.6630271**	**2.124045267**	**344.8600171**	**0.956453879**	**344.8665582**
ASTE006568	195.5610393	27.78994462	2.814984113	167.7710947	0.959575464	167.7947089
ASTE014971	40.68745778	11.64369631	1.805035025	29.04376147	0.951108163	29.0997978
ASTE002870	108.1619625	8.932482199	3.59798829	99.22948032	0.984118932	99.2946891
ASTE002872	960.8634615	137.9330178	2.800363595	822.9304437	0.959497425	822.9352084
ASTE003164	451.8315868	67.44145097	2.74407765	384.3901358	0.959380365	384.3999303
ASTE010888	233.441121	43.59729117	2.420748312	189.8438298	0.95836585	189.859263

ASTE011055	57.43423605	16.388321	1.809242915	41.04591505	0.952786015	41.08577006
ASTE010909	42.09114583	1.936581507	4.441932656	40.15456433	0.9911425	40.3995025
ASTE008793	80.06870585	16.7756373	2.254870913	63.29306855	0.957117216	63.33322169
ASTE005244	94.20306469	23.11794174	2.02676104	71.08512295	0.954776026	71.11401033
ASTE003772	465.1471276	7.020107962	6.05005009	458.1270196	0.99996098	458.1669665
ASTE003443	43.02693787	11.9583908	1.847216906	31.06854706	0.95173248	31.12341284
ASTE003899	1313.754536	387.921483	1.759859166	925.8330534	0.950132667	925.834726
ASTE016029	53.32065023	9.513456651	2.486652839	43.80719358	0.957741533	43.87771247
ASTE016031	32.20684248	2.711214109	3.570356273	29.49562837	0.981894803	29.71093295
ASTE010780	43.45584255	12.17625622	1.835479535	31.27958632	0.95173248	31.33339282
ASTE010806	362.2879866	36.33511052	3.317700767	325.952876	0.972295926	325.9697602
ASTE003809	9.45539867	0.145243613	6.02459166	9.310155057	0.968745123	11.08939547
ASTE005482	169.378358	49.5522793	1.773226224	119.8260787	0.950132667	119.8391984
ASTE006972	947.7428774	28.39512634	5.060780473	919.3477511	0.996722335	919.3616801
ASTE006979	57.1028097	2.783835916	4.35841565	54.31897378	0.989425628	54.49354732
ASTE006284	192.3247585	37.5938885	2.354974445	154.73087	0.957507414	154.7487901
ASTE001820	121.6919557	25.92598492	2.2307631	95.76597075	0.957195255	95.79194881
ASTE014603	88.64679949	23.91678161	1.89004528	64.73001788	0.953605432	64.75760563
ASTE016303	762.4755505	139.2160031	2.453366017	623.2595474	0.95840487	623.2643761
ASTE016305	540.8293083	130.8887026	2.046832756	409.9406057	0.955595442	409.9457156
ASTE016306	6378.884882	1881.340519	1.761543245	4497.544363	0.950132667	4497.544708
ASTE016302	1498.242037	112.3217274	3.737561773	1385.920309	0.984118932	1385.925349
ASTE008686	82.73961228	8.133642328	3.346604741	74.60596995	0.972295926	74.68099166
ASTE016150	1120.182055	181.264029	2.62756866	938.9180262	0.959341345	938.9217028
ASTE003419	39.79065708	4.35730839	3.190920594	35.43334869	0.965194319	35.57673641
ASTE015911	60.14413381	15.46844478	1.959095876	44.67568902	0.953722491	44.71862304
ASTE009307	133.0189384	19.3416078	2.781852031	113.6773306	0.959497425	113.7113635
ASTE006624	317.486943	3.582675787	6.469515957	313.9042672	1	313.9709279
ASTE005675	137.0545415	34.08383452	2.007590579	102.970707	0.954424848	102.9902759
ASTE000995	923.1003539	255.6529661	1.852300733	667.4473877	0.953644451	667.449958
ASTE000319	13.29604514	0.750425334	4.147144842	12.54561981	0.969057281	13.2133034
ASTE005986	85.2935447	22.58538182	1.917047254	62.70816288	0.953566412	62.737459
ASTE006025	20494.50838	4277.037086	2.260553761	16217.4713	0.957234275	16217.47145
ASTE007977	8.207675959	0.338901764	4.598034724	7.868774195	0.95165444	9.113700163
ASTE006671	300.662182	63.64090976	2.240117049	237.0212723	0.957234275	237.0318579
ASTE006676	94.32003869	5.882366326	4.003095774	88.43767237	0.984118932	88.52822527
ASTE014742	9.338424666	0.266279957	5.13216345	9.072144709	0.965818636	10.42319103
ASTE001076	618.2855947	169.9592345	1.863084642	448.3263602	0.953644451	448.3302313
ASTE016138	111.0863126	12.15204895	3.192409576	98.93426367	0.966520993	98.98575658
ASTE009073	32.12885981	4.502552003	2.835054939	27.62630781	0.956453879	27.77139571
ASTE005433	64.94006798	10.84485644	2.58209794	54.09521154	0.959068207	54.15680143
ASTE009198	9.669851011	0.508352645	4.249592111	9.161498366	0.955439363	10.0991131

ASTE006822	139.7449436	7.092729768	4.300311291	132.6522139	0.989698767	132.7218992
ASTE006824	100.9095743	8.448336823	3.578251899	92.46123744	0.984118932	92.53045074
ASTE006910	78.08014778	18.78484061	2.055386908	59.29530717	0.955361324	59.33091999
ASTE000047	63.6728496	12.15204895	2.389478745	51.52080065	0.957897612	51.5761816
ASTE008066	208.7791018	42.07223323	2.311037004	166.7068685	0.957468394	166.7228866
ASTE000499	34.35136589	6.366511703	2.431792443	27.98485419	0.955400343	28.09031289
ASTE006794	124.1094184	24.25568337	2.355217872	99.85373505	0.957819572	99.88150707
ASTE004605	107.3041532	27.06372656	1.987273505	80.2404266	0.954424848	80.26503172
ASTE015132	819.7343255	101.4768709	3.014005471	718.2574546	0.959770563	718.2637783
ASTE009889	35.07270558	6.197060821	2.500692695	28.87564476	0.955985641	28.98372516
ASTE008151	88.51032982	8.278885941	3.418337291	80.23144388	0.978304979	80.30423162
ASTE008162	11.61941775	0.944083484	3.621479525	10.67533426	0.959965663	11.27288231
ASTE008166	61.54782186	16.87246638	1.867036923	44.67535548	0.952981114	44.71435132
ASTE006152	389.6599035	72.42814835	2.427593086	317.2317552	0.95836585	317.2410435
ASTE006153	266.7202252	53.5948932	2.315159783	213.125332	0.957468394	213.1379063
ASTE011469	8.012719285	0.048414538	7.370707807	7.964304748	0.958678008	10.85161203
ASTE005296	417.0903075	75.47826422	2.466226636	341.6120433	0.95840487	341.6209455
ASTE005321	81.04348922	22.39172367	1.855730701	58.65176555	0.953410332	58.68111569

Table S3 List of upregulated genes identified through GFOLD

GeneSymbol	GFOLD(0.01)	E-FDR	log2fdc	1stRPKM (Che-s)	2ndRPKM (Alw-R)
ASTE000047	1.95019	1	2.11679	5.11494	26.0051
ASTE000319	3.28782	1	3.84687	1.57596	27.3465
ASTE000499	1.92811	1	2.16237	3.77158	19.8289
ASTE000995	1.57534	1	1.61239	127.204	455.094
ASTE001076	1.57827	1	1.62222	134.051	482.887
ASTE001820	1.89104	1	2.00362	6.15155	28.8855
ASTE001895	2.26501	1	2.40934	15.473	96.3141
ASTE002155	2.93399	1	3.43709	1.82411	23.6572
ASTE002156	3.91874	1	3.98117	29.1652	539.07
ASTE002870	2.50648	1	2.57507	106.223	740.845
ASTE002872	2.50652	1	2.55465	35.1003	241.321
ASTE003164	2.43266	1	2.50278	24.6169	163.3
ASTE003419	2.72568	1	2.99323	1.79583	16.8271
ASTE003443	1.40614	1	1.57599	2.46956	8.62882
ASTE003772	5.68656	1	5.89049	3.61393	251.801
ASTE003809	4.29207	1	5.55404	0.284911	17.8662
ASTE003899	1.49265	1	1.52195	619.341	2081.1

ASTE004102	1.73373	1	1.86886	5.76526	24.6654
ASTE004212	1.54784	1	1.58692	33.4616	117.621
ASTE004605	1.65228	1	1.76717	11.8066	47.0576
ASTE005244	1.66365	1	1.78521	5.19686	20.9761
ASTE005296	2.18439	1	2.251	30.851	171.873
ASTE005321	1.47733	1	1.60821	11.958	42.6968
ASTE005433	2.12483	1	2.31088	5.89019	34.2785
ASTE005482	1.438	1	1.52357	8.57269	28.8485
ASTE005675	1.64013	1	1.76309	51.2604	203.757
ASTE005696	2.13298	1	2.21353	65.9338	357.953
ASTE005881	1.53081	1	1.62705	18.7197	67.6881
ASTE005891	1.93998	1	2.015	45.373	214.643
ASTE005986	1.59137	1	1.72009	8.1805	31.5644
ASTE006025	2.02433	1	2.03367	2481.48	11887.7
ASTE006152	2.12127	1	2.1982	150.742	809.691
ASTE006153	2.00834	1	2.0855	82.8873	411.759
ASTE006192	1.70732	1	1.75815	57.8666	229.055
ASTE006284	2.01409	1	2.10936	12.8323	64.8239
ASTE006325	3.08001	1	3.13643	56.8659	585.202
ASTE006367	2.78785	1	2.90602	8.86977	77.8688
ASTE006568	2.46289	1	2.5728	6.93789	48.3376
ASTE006624	5.93558	1	6.20394	2.04945	177.981
ASTE006671	1.91241	1	1.98692	31.1222	144.39
ASTE006676	3.62203	1	3.85339	2.47653	42.0759
ASTE006794	1.97923	1	2.10661	15.7631	79.5158
ASTE006822	3.90692	1	4.10796	3.3228	67.2734
ASTE006824	3.19764	1	3.38624	2.76014	33.8648
ASTE006910	1.69981	1	1.83338	3.56413	14.8773
ASTE006972	4.72131	1	4.82523	8.63775	286.773
ASTE006979	3.75905	1	4.06809	1.36751	27.0689
ASTE007704	3.41065	1	3.70134	1.59499	24.4549
ASTE007977	3.47254	1	4.44059	0.244417	6.75651
ASTE008066	1.99389	1	2.08278	38.6121	191.476
ASTE008151	2.98651	1	3.17903	2.14571	22.8056
ASTE008162	2.75962	1	3.27086	0.0808607	0.934931
ASTE008166	1.5128	1	1.65777	7.29114	26.95
ASTE008531	6.31558	1	7.67615	0.044576	12.7932
ASTE008686	2.92383	1	3.11431	3.97092	40.3498

ASTE008793	1.88014	1	2.0378	28.603	137.641
ASTE008971	4.51268	1	4.82019	3.78702	126.283
ASTE009073	2.39047	1	2.66357	2.17988	16.2528
ASTE009198	3.16584	1	3.84371	0.483897	8.51569
ASTE009307	2.41125	1	2.53817	6.42844	43.7386
ASTE009456	2.45864	1	2.50984	440.14	2933.57
ASTE009555	1.99092	1	2.11362	29.7774	150.928
ASTE009889	1.81049	1	2.04129	9.0065	43.5297
ASTE010010	3.14965	1	3.40023	3.69711	45.9105
ASTE010150	1.81331	1	1.8853	13.9428	60.2851
ASTE010348	1.99396	1	2.23821	1.89175	10.4869
ASTE010406	2.05213	1	2.10232	287.828	1446.29
ASTE010525	1.60308	1	1.71359	4.23253	16.2535
ASTE010606	1.93808	1	2.2024	0.961005	5.20039
ASTE010780	1.46666	1	1.6334	9.61745	34.9671
ASTE010806	2.9379	1	3.03008	19.1432	183.077
ASTE010888	2.06251	1	2.15446	11.9774	62.4243
ASTE010909	3.8201	1	4.19101	1.56283	33.8266
ASTE011055	1.4729	1	1.62234	4.68043	16.8815
ASTE011469	4.81909	1	6.44059	0.0967794	14.7142
ASTE011694	1.80307	1	1.8696	46.8584	200.402
ASTE014603	1.58904	1	1.71662	37.6667	144.985
ASTE014742	4.10597	1	5.06529	0.182382	7.78083
ASTE014827	2.48384	1	2.88983	8.71015	76.5727
ASTE014971	1.44997	1	1.6267	15.7825	57.1278
ASTE015132	2.7974	1	2.85234	110.478	933.688
ASTE015911	1.59873	1	1.74358	10.6913	41.9412
ASTE016029	1.86997	1	2.06372	8.71887	42.7543
ASTE016031	2.95862	1	3.29823	2.34153	27.2165
**ASTE016034 (AsGSTe2)**	**1.72944**	**1**	**1.81215**	**75.7626**	**311.412**
**ASTE016035 (AsGSTe4)**	**1.86215**	**1**	**1.9236**	**134.33**	**596.384**
ASTE016138	2.78032	1	2.94331	12.8467	115.852
ASTE016150	2.33843	1	2.3809	59.5648	363.04
ASTE016243	1.57001	1	1.66592	2.10918	7.83482
ASTE016302	3.40903	1	3.45817	140.3	1804.44
ASTE016303	2.15484	1	2.20249	176.883	952.701
ASTE016305	1.80243	1	1.85108	181.359	765.63

ASTE016306	1.4887	1	1.50213	2416.5	8008.75
ASTE016435	1.75865	1	1.78361	542.295	2184.55
ASTE016494	1.52425	1	1.60335	19.0639	67.7972

Figure S1

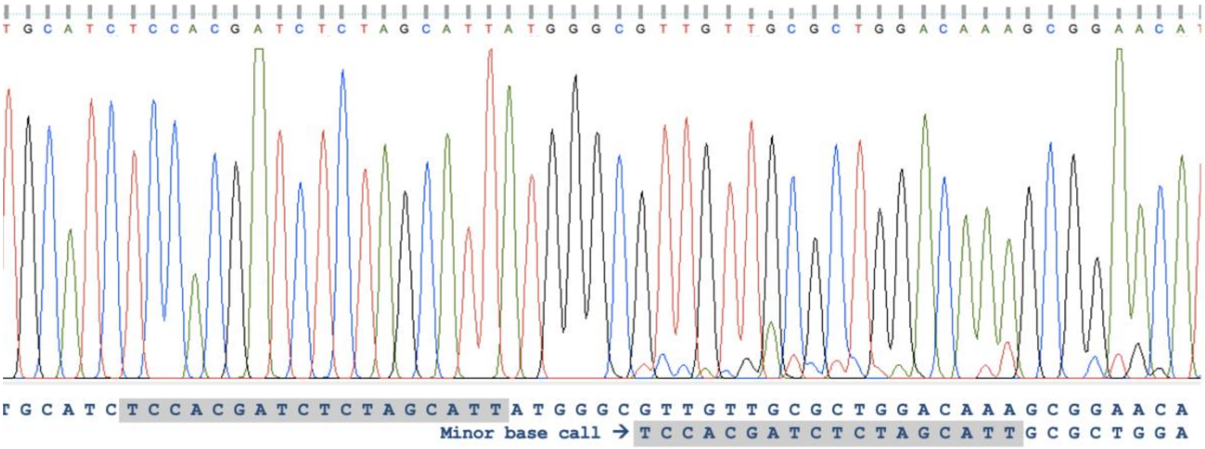

Figure S1: Snapshot of DNA sequence chromatogram showing disproportionate peak heights of variants *AsGSTe2.1* and *AsGSTe2.1* (residual peaks) in a mosquito. The first line of sequence shown at the bottom of the chromatogram is nucleotide base call of major variant *AsGSTe2*.1 and the second line is base call of residual peak of variant *AsGSTe2.2* appearing from the point of indel. The highlighted sequence blocks show repeat motif in *AsGSTe2.2*.

```
PS2.1(1)      CATGGAAGTCTTTAAATAAAGAGGCACAAACGTAACTTGAAGCAGCTTCCTATTGAAGTTATCTTCTCGATTAATCATCA   80
PS2.1(2)      ................................................................................
PS2.1(3)      ................................................................................
Afghanistan                                   .......................G........................
PS2.2         ................................................................................
Delhi         ................................................................................
JQ031268                                      ................................................
KB664467      ..----................A.........................................................
CP032234      ..----................A.........................................................
Chennai       ..----................A.........................................................

PS2.1(1)      GTTGGCTTTCTTCACATTTGTACTTGTGTGTTTCGTACTTGGTACTGGATGCGAACTGAGTTGTACTTGCTGCGGGGCAG  160
PS2.1(2)      ................................................................................
PS2.1(3)      ................................................................................
Afghanistan   ................................................................................
PS2.2         ................................................................................
Delhi         ................................................................................
JQ031268      ................................................................................
KB664467      ................................-...G...........................................
CP032234      ................................-...G...........................................
Chennai       ................................-...G...........................................

PS2.1(1)      AAATTGGGACCATCATTAGCGACCGTCAGGCCATCACAG-----------------------CTTGGAGCTGGTCAACTC  240
PS2.1(2)      .......................................-----------------------..................
PS2.1(3)      .......................................-----------------------..................
Afghanistan   .......................................-----------------------...................
PS2.2         .........................G...A.........GGCAGTTGAAGGAATCTAATGGTG.............G..T
Delhi         .........................G...A.........GGCAGTTGAAGGAATCTAATGGTG.............G..T
JQ031268      .........................G...A.........GGCAGTTGAAGGAATCTAATGGTG.............G.CT
KB664467      .......................................GGCAGTTGAAGGAATCTAATAGTG..........G..G...
CP032234      .......................................GGCAGTTGAAGGAATCTAATAGTG..........G..G...
Chennai       .......................................GGCAGTTGAAGGAATCTAATAGTG..........G..G...

PS2.1(1)      TGGCGTGTCTTTCCAACGCAACGATTGCTTCAAAATGTCTAACTTGGTTCTGTACACGCTTGAACCCACAGTGACACGCT  320
PS2.1(2)      ................................................................................
PS2.1(3)      ................................................................................
Afghanistan   ................................................................................
PS2.2         ......................................A...G..A.......................T....A.....
Delhi         ......................................A...G..A.......................T....A.....
JQ031268      ......................................A...G..A.......................T....A.....
KB664467      .......G.....................................A.......................T........T.
CP032234      .......G.....................................A.......................T........T.
Chennai       .......G.....................................A.......................T........T.

PS2.1(1)      GTAGAGTTAACAGGAAACATACCTGTTTGCAGGTGGTCATATGAAGCCGGAGTTCCTGCAGCTATAGGTCCTTGCCGATG  400
PS2.1(2)      ................................................................................
PS2.1(3)      ................................................................................
Afghanistan   ................................................................................
PS2.2         ...................G.........................................G............T....A
Delhi         ...................G.........................................G............T....A
JQ031268      ...................G.........................................G............T....A
KB664467      ...................G.........................................G..................
CP032234      ...................G.........................................G..................
Chennai       ...................G.........................................G..................

PS1 (1)       TCCATTATGGTGGTATTTTCTATTGAGCGATCGTTATAGCTTAACCCTCAACATGCGAACCTGGTGCTGGATGATGATGG  480
PS1 (2)       ................................................................................
PS1 (3)       ................................................................................
Afghanistan   ................................................................................
PS2           .........................G..........C.....................T..................A..
Delhi         .........................G..........C.....................T..................A..
JQ031268      .........................G..........C.....................T..................A..
KB664467      ....................................C.................A...T..................A..
CP032234      ....................................C.................A...T..................A..
Chennai       ....................................C.................A...T..................A..

PS2.1(1)      TACGATCATCACCGAGAGTCATGCGATCATGATCTATCTGGTGCCAGTCTTAACACGACAGGACCGGGGTTCAAATCGCA  560
PS2.1(2)      ................................................................................
PS2.1(3)      ................................................................................
Afghanistan   ................................................................................
PS2.2         ............................------.........T.............G.............A........
Delhi         ............................------.........T.............G.............A........
JQ031268      ............................------.........T.............G.............A........
KB664467      .........................................................G.............A.....T..
CP032234      .........................................................G.............A.....T..
Chennai       .........................................................G.............A.....T..
```

```
PS2.1(1)    TCTAAGCCGTTCCCCCCATAGTGAGGCCAAGACTTCTCGAGGTTGTA------AGGAAGAAGAAGAAGGAACCGATTGAG   640
PS2.1(2)    ...............................................------...........................
PS2.1(3)    ...............................................------...........................
Afghanistan ...............................................------...........................
PS2.2       ..C............................................GTGCCA........................T..
Delhi       ..C............................................GTGCCA........................T..
JQ031268    ..C............................................GTGCCA........................T..
KB664467    .................................C.............------........................T..
CP032234    .................................C.............------........................T..
Chennai     .................................C.............------........................T..

PS2.1(1)    GGAAGT--TCACATTTGCCAAGAAGGAAGAAAATGGTGAAGTTTTTAACTCAAATGCGATTTTTATGACCGAAAAACACC   720
PS2.1(2)    ......--...................................................................-....
PS2.1(3)    ......--........................................................................
Afghanistan ......--...................................................................-....
PS2.2       ......GT...T.............A...............C............A...................---...
Delhi       ......GT...T.............A...............C............A...................---...
JQ031268    ......GT...T.............A...............C............A...................---...
KB664467    ......GT...T.............................C................................---...
CP032234    ......GT...T.............................C................................---...
Chennai     ......GT...T.............................C................................---...

PS2.1(1)    AGTACATCAGGAGTATAACAGAAACAGTTTAAATCTTATCATGCACTGATAGCTAGACAATAAAACGAGAAGTAAGTGGC   800
PS2.1(2)    .........A...................-..................................................
PS2.1(3)    .............................-..................................................
Afghanistan .............................-..................................................
PS2.2       .........A.....A.............-..................................................
Delhi       .........A.....A.............-.............................-....................
JQ031268    .........A.....A.............-..................................................
KB664467    .........A.....A.............-............AT......................A.............
CP032234    .........A.....A.............-............AT......................A.............
Chennai     .........A.....A.............-............AT......................A.............

PS2.1(1)    TATTAAAAAAGAAAATTTGATACAAGTCGGAATCAAGGTGCATGTAAAGAAACGCGAAAGAAAGACGAACAAATTGTTAA   880
PS2.1(2)    ................................................................................
PS2.1(3)    ................................................................................
Afghanistan .......................................................T..........A.......C.....
PS2.2       .........T.............................................T..........A.......C.....
Delhi       .........T.................T...........................T..........A.......C.....
JQ031268    ................................................................................
KB664467    .........T.............................................T..........A.......C.....
CP032234    .........T.............................................T..........A.......C.....
Chennai     .........T.............................................T..........A.......C.....

PS2.1(1)    AGTTGACAAAACC   893
PS2.1(2)    .............
PS2.1(3)    .............
Afghanistan .............
PS2.2       .............
Delhi       .............
JQ031268    .............
KB664467    .............
CP032234    .............
Chennai     .............
```

Figure S2 Alignment of nucleotide sequences of *ψAsGSTe2*. PS2.1and PS2,2 represent pseudogenes *ψAsGSTe2.1* and *ψAsGSTe2.2*, respectively, present in Alw-R. KB66446. CP032234 represent *ψAsGSTe2* sequences of SDA-500 strain (originating Pakistan). Delhi and Chennai represent the locality of strains having a single copy of *ψAsGSTe2*. Sequences of Beech (JQ031268, origin: Delhi) and Afghanistan were sourced from Ayres et al [1].

[1] Ayres CF, Müller P, Dyer N, Wilding CS, Rigden DJ, Donnelly MJ. Comparative genomics of the anopheline glutathione S-transferase epsilon cluster. PLoS ONE 2021; 6: e29237. doi:10.1371/journal.pone.0029237
